# Assessment of absolute abundance in mother-infant gut microbiome using marine-sourced bacterial DNA spike-in and comparison with conventional quantification methods

**DOI:** 10.20517/mrr.2024.94

**Published:** 2025-06-09

**Authors:** Shuo Wang, David Healy, Dhrati Patangia, Shona Uniacke-Lowe, Elena Kamilari, Iwona M. Kozak, Bo Yang, Eugene M. Dempsey, Catherine Stanton, R. Paul Ross

**Affiliations:** ^1^APC Microbiome Ireland, University College Cork, Cork T12 YT20, Ireland.; ^2^Department of Paediatrics and Child Health, University College Cork, Cork T12 YT20, Ireland.; ^3^Teagasc Food Research Centre, Moorepark, Fermoy P61 C996, Ireland.; ^4^INFANT Research Centre, University College Cork, Cork T1 DC4A, Ireland.; ^5^School of Food Science and Technology, Jiangnan University, Wuxi 214122, Jiangsu, China.

**Keywords:** Spike-in, absolute quantification, marine-sourced bacteria

## Abstract

**Aim:** To evaluate the effectiveness of marine-sourced bacterial DNA spike-in quantification for determining absolute microbial abundance in the gut microbiome of mother-infant pairs and to compare this method with conventional quantification techniques.

**Methods:** We conducted a pilot study involving six mother-infant pairs, applying a DNA spike-in quantification method using bacterial DNA from *Pseudoalteromonas* sp. APC 3896 and *Planococcus* sp. APC 3900, isolated from deep-sea fish. We compared our approach with established absolute quantification methods - flow cytometry, total DNA measurement, quantitative PCR (qPCR), and culture-based plate count - to evaluate microbial load and taxonomic composition across mother-infant samples.

**Results:** Our spike-in method accurately estimated microbial loads, producing results consistent with qPCR and total DNA quantification. We observed that mothers exhibited higher total bacterial loads than infants by approximately half a log, while the abundance of *Bifidobacterium* was comparable in both groups. The spike-in method revealed significant differences in taxonomic composition, highlighting the impact of absolute quantification on microbiome analysis outcomes. Importantly, the method did not alter alpha diversity measures but slightly affected beta diversity analysis, reflecting more precise inter-group differences.

**Conclusion:** Marine-sourced bacterial DNA spike-in offers a reliable, scalable, and accurate approach for absolute microbiome quantification. This method enhances microbiome analysis by addressing biases inherent in relative abundance measures, providing a deeper understanding of microbial dynamics in mother-infant gut microbiomes.

## INTRODUCTION

Relative abundance measurements rely on normalizing sequencing data to account for variations in sequencing depth, sample composition, and other technical factors^[1]^. However, normalization methods may introduce biases and inaccuracies, particularly when dealing with complex microbial communities or samples with low biomass^[2,3]^. When examining relative abundance data, an increase in the abundance of one taxon results in a corresponding decrease in the abundance of other taxa. Consequently, assessing the relative abundance of a taxon depends on the abundance of all other taxa, potentially leading to elevated false-positive rates in differential taxon analyses and negative correlation biases in correlation-based analyses^[4,5]^.

Absolute Quantification methods offer a more accurate approach by directly measuring the absolute abundance of microbial taxa within a sample. Techniques such as flow cytometry^[6]^, qPCR^[7-9]^, machine-learning^[10]^, and total DNA^[11,12]^ quantification have been utilized for absolute quantification but may face limitations, including issues with specificity, sensitivity, and scalability. For instance, flow cytometry-based techniques necessitate the sample’s dissociation into individual bacterial cells, often involving intricate sample preparation^[13]^. In addition, bacterial suspensions must be diluted to optimal concentrations (typically 10^5^-10^7^ cells/mL) to avoid coincidence artifacts and ensure accurate event detection^[14]^. Achieving this range can be technically challenging in low-biomass or small-volume samples, such as infant feces, potentially compromising measurement accuracy. Total DNA-based approaches are confounded by the presence of host DNA, particularly in low-biomass samples such as infant feces. qPCR provides taxonomic specificity but is subject to primer-dependent amplification bias, which may disproportionately affect the quantification of dominant taxa such as *Bifidobacterium* in infant gut samples^[15]^. These limitations are especially consequential in mother-infant microbiome studies, where sample volumes are typically small, host DNA contamination is common, and microbial composition differs markedly from adults.

The spike-in method has also been employed in microbial absolute quantification, mainly in two forms: either by directly introducing exogenous cells into fecal samples^[16,17]^ or by adding synthetic DNA to the sample DNA^[18,19]^. Corresponding reagent kits have already been developed and are commercially available^[19,20]^. However, to date, no published studies have explored the use of marine bacterial DNA as a spike-in for absolute quantification in sample DNA.

To address these challenges, reliable methods are needed that offer substantial reproducibility and accurately provide the overall absolute abundance across both high- and low-density samples. Additionally, the method should be easily applicable to high throughput workflows and be cost-effective for large-scale studies.

In this study, we compared various approaches for absolute microbiome quantification, including a DNA spike-in strategy in which exogenous bacterial DNA is added to sample DNA to enable accurate estimation of absolute bacterial abundances. We selected *Pseudoalteromonas* sp. APC 3896 and *Planococcus* sp. APC 3900-marine strains isolated from deep-sea fish that represent phylogenetically distinct phyla rarely found in mammalian fecal microbiomes. These genera are evolutionarily distant from gut-associated microbes and are absent not only at the species level but also at the genus level in the human gut microbiome under typical physiological conditions. Moreover, they are easily distinguishable from endogenous bacteria through 16S rRNA gene sequencing. Their use is also technically convenient, as well-characterized isolates were readily available in our laboratory.

The other absolute quantification approaches, including flow cytometry, total DNA quantification, qPCR, and plate count, were compared with the DNA spike-in quantification. We then applied the method to quantify absolute bacterial counts in the feces obtained from infants and their mothers, which highlighted the difference in microbial analysis between relative abundance and absolute abundance.

## METHODS

### Participant recruitment

Stool samples were obtained from mothers (*n* = 6) and their infants (*n* = 6) enrolled in the MIMIC study^[21]^. All samples were collected at 4 weeks after birth. The protocol was approved by the Clinical Research Ethics Committee of the Cork Teaching Hospitals and has been previously published^[21]^. Inclusion criteria included full-term infants (gestational age ≥ 37 weeks) and availability of paired maternal and infant fecal samples within the first month postpartum. Exclusion criteria were antibiotic use by the infant within 2 weeks prior to sample collection, neonatal complications requiring intensive care, or any known gastrointestinal disorders.

### Flow cytometry

For flow cytometry (BD FACSCelesta, BD Science, USA) analysis, 0.05 g aliquots of fecal samples were diluted 10,000-fold in 0.85% NaCl. This dilution ensured that bacterial concentrations fell within the optimal range for accurate detection (10^5^-10^7^ cells/mL), minimized background noise, and maintained compatibility with viability dyes and microsphere calibration. To remove debris from the fecal solutions, samples were filtered using sterile syringe filters with a pore size of 5 μm. The LIVE/DEAD**^TM^** BacLight**^TM^** Bacterial Viability and Counting Kit (Invitrogen, USA) was used to distinguish and quantify live and dead bacteria. This kit utilizes a mixture of two nucleic acid stains: green-fluorescent SYTO**^TM^** 9 dye and red-fluorescent propidium iodide, for viability determinations. Additionally, a calibrated suspension of microspheres was employed for accurate sample volume measurements.

### Plate count

For the quantification of viable bacterial populations, fresh stool samples from mothers and infants were subjected to plate count on YCFA (Yeast Extract, Casitone, Fatty Acids) medium^[22]^. Stool samples (~ 1 g) were homogenized in 9 mL of sterile phosphate-buffered saline (PBS) to prepare a 1:10 dilution, followed by serial dilutions to ensure countable colony formation. Aliquots (100 µL) of each dilution were plated onto YCFA agar plates and spread evenly using sterile spreaders. Plates were immediately transferred into a Whitley A20 anaerobic workstation (Don Whitley Scientific Limited, UK) and incubated at 37 °C for 48 h. After incubation, colonies on plates containing 30-300 colony-forming units (CFUs) were enumerated, and bacterial counts were calculated as CFUs per gram of stool. Negative controls with sterile PBS and YCFA plates were included to monitor contamination.

### Spike-in bacteria and quantification

In this study, we utilized *Planococcus* sp. APC 3900 (NCBI: txid3035191) and *Pseudoalteromonas* sp. APC 3896 (NCBI: txid3035187), isolated from the skin of deep-sea fish, as previously described^[23]^. These marine bacteria, belonging to the Pseudomonadota and Bacillota phyla, respectively, are typically absent from mammalian fecal microbiomes under normal physiological conditions. They are easily distinguishable from bacteria commonly found in the gut using 16S rRNA gene sequencing^[24,25]^. These strains are effectively amplified by standard V3-V4 16S rRNA primers, enabling their quantification during sequencing. While 16S rRNA sequencing typically resolves taxa at the genus level, the absence of *Pseudoalteromonas* and *Planococcus* genera in human gut microbiomes allows genus-level abundance to reliably represent these spike-in strains. The strains were cultured in DifcoTM 2216 marine broth (BD DifcoTM, New Jersey, USA) and incubated aerobically with agitation at 30 °C for 24 h. *Pseudoalteromonas* sp. APC 3896 is a gram-negative bacterium, whereas *Planococcus* sp. APC 3900 is a gram-positive bacterium. The 16S rRNA gene copy numbers per genome for the spike-in bacteria were obtained from the rrnDB database^[26]^. The copy numbers of the spike-in bacteria were calculated using the formula: number of copies (molecules) = (amount of DNA ng × 6.022 × 10^23^ molecules/mole) / (length of dsDNA amplicon × 660 g/mole × 1 × 10^9^ ng/g).

### DNA extraction

Genomic DNA was extracted from the mother and infant samples using the QIAmp Mini stool DNA extraction kit (Qiagen, USA), following the manufacturer’s instructions with slight modifications, as recommended in prior studies^[27]^. Approximately 0.2 g of stool sample was transferred into a tube containing around 50 mg of pre-sterilized zirconia beads of various sizes (Biospec, USA), followed by the addition of 1ml of lysis buffer. The mixture was then subjected to bead beating for homogenization, and nucleic acid extraction was performed according to the manufacturer’s instructions. The concentration of sample DNA and spike-in DNA was measured using the Qubit**^TM^** 1X dsDNA High Sensitivity (HS) assay kit and Qubit 4 (Invitrogen, USA).

### qPCR assessment of bacterial loads

DNA extracted from fecal samples served as a template for qPCR amplification of bacterial 16S rRNA genes using the primer pair^[28]^ U16SRT-F (ACTCCTACGGGAGGCAGCAGT) and U16SRT-R (TATTACCGCGGCTGCTGGC) on an Applied Biosystems 7500 Fast Real-Time PCR System (Thermo Fisher Scientific). Another primer pair F-bifido (CGCGTCYGGTGTGAAAG) and R-bifido (CCCCACATCCAGCATCCA) specific for the genus *Bifidobacterium* was also used^[29]^. PCR assays were prepared with 10 μL PowerUp SYBR Green Master Mix (Thermo Fisher Scientific), 0.8 μL of each primer solution (10 μm), 7.4 μL of sterile nuclease-free water, and 1 μL of template DNA solution. The amplification program included initial denaturation at 95 °C for 180 s, followed by 40 cycles of denaturation at 95 °C for 10 s, annealing at 60 °C for 30 s, and extension at 72 °C for 30 s. Each run included negative controls (without DNA), extraction controls (without pellet), and positive controls (with genomic DNA from *Pseudoalteromonas* sp. APC 3896 and *Planococcus* sp. APC 3900). A melting curve analysis was conducted after each run, and cycle threshold values were determined using 7500 software v2.0.6 (Thermo Fisher Scientific). All qPCR assays were performed in triplicate. The standard curve was employed to convert the Ct values obtained from the qPCR run into absolute copy numbers. This curve establishes a linear relationship between Ct values and the logarithm of the initial DNA concentration. By correlating the Ct values of the samples with the standard curve, the total copy number of the DNA in each stool sample can be accurately determined.

### Library preparation

Twenty microliters of fecal sample DNA were mixed evenly with 2.5 µL of DNA from *Pseudoalteromonas* sp. APC 3896 (4.31 ng/µL) and 2.5 µL of DNA from *Planococcus* sp. APC 3900 (9.59 ng/µL). The DNA concentration was quantified using Qubit 4 and normalized to 5 ng/μL with 10 mm Tris before library preparation. In this study, 2.32 × 10^6^ copies of *Pseudoalteromonas* sp. APC 3896 and 5.99 × 10^6^ copies of *Planococcus* sp. APC 3900 were added to achieve a final relative abundance of the spike-in between 0.1%-10% in most samples. The DNA samples were amplified, targeting the V3 and V4 regions of the 16S rRNA gene using primers 341F/806R. This process produced an amplification fragment of approximately 465 bp. Following the manufacturer's instructions, the amplicons were prepared for sequencing and analyzed using the Illumina HiSeq 2000 platform with 2 × 250 bp chemistry (Illumina Technologies, USA).

### Computational analysis

The raw paired-end reads were analyzed using the dada2 pipeline. Denoising and pre-processing of the sequence reads were performed using the dada2 pipeline^[30]^, while filtered and trimmed sequence data were processed using the core sample inference algorithm. Taxonomy assignment to the Amplicon Sequence Variants (ASVs) was conducted using the SILVA database release 138.1^[31]^. Diversity analysis was performed in R using phyloseq and microbiome package.

### Statistical analysis

SPSS 25.0 (Stanford, CA, USA) was used to perform statistical testing. The data were all tested for normal distribution before comparison^[32]^. For comparing the two groups of data, the Shapiro-Wilk test was used to assess normality^[33]^. If the data followed a normal distribution (*P* > 0.05), an independent sample t-test was employed. If the data did not conform to a normal distribution (*P* < 0.05), the non-parametric Mann-Whitney *U* test was used^[34]^. For comparisons involving three or more groups, normality and homogeneity of variances were first tested. If these assumptions were met, one-way ANOVA followed by Post hoc Tukey’s test was used. If the data did not meet normality or homogeneity of variance assumptions, the non-parametric Kruskal-Wallis test was applied^[35]^. For correlation analysis, the Shapiro-Wilk test was used to assess normal distribution. If the data were normally distributed, the Pearson correlation coefficient was used; otherwise, the Spearman correlation coefficient was used^[36]^.

## RESULTS

Given the increasing need for accurate quantification of microbial abundances in microbiome studies, particularly in relation to 16S rRNA and metagenomic sequencing outputs, we evaluated various approaches to establish a reliable method for absolute quantitation. Traditional relative abundance measures, while useful, often fail to capture the true variability in microbial load, leading to potential misinterpretations of microbiome dynamics. To address this limitation, we implemented a DNA spike-in quantification method that enables the determination of absolute microbial counts, providing a more comprehensive view of microbial shifts and interactions. In this study, we compared the performance of our spike-in method against conventional absolute quantification techniques such as flow cytometry, total DNA quantification, qPCR, and plate count assessment. This approach allowed us to explore differences in microbial loads between mothers and infants and examine how absolute quantification can alter microbiome analysis outcomes, thereby emphasizing the necessity for absolute measures in microbiome research.

### Experimental design

**Figure 1:**
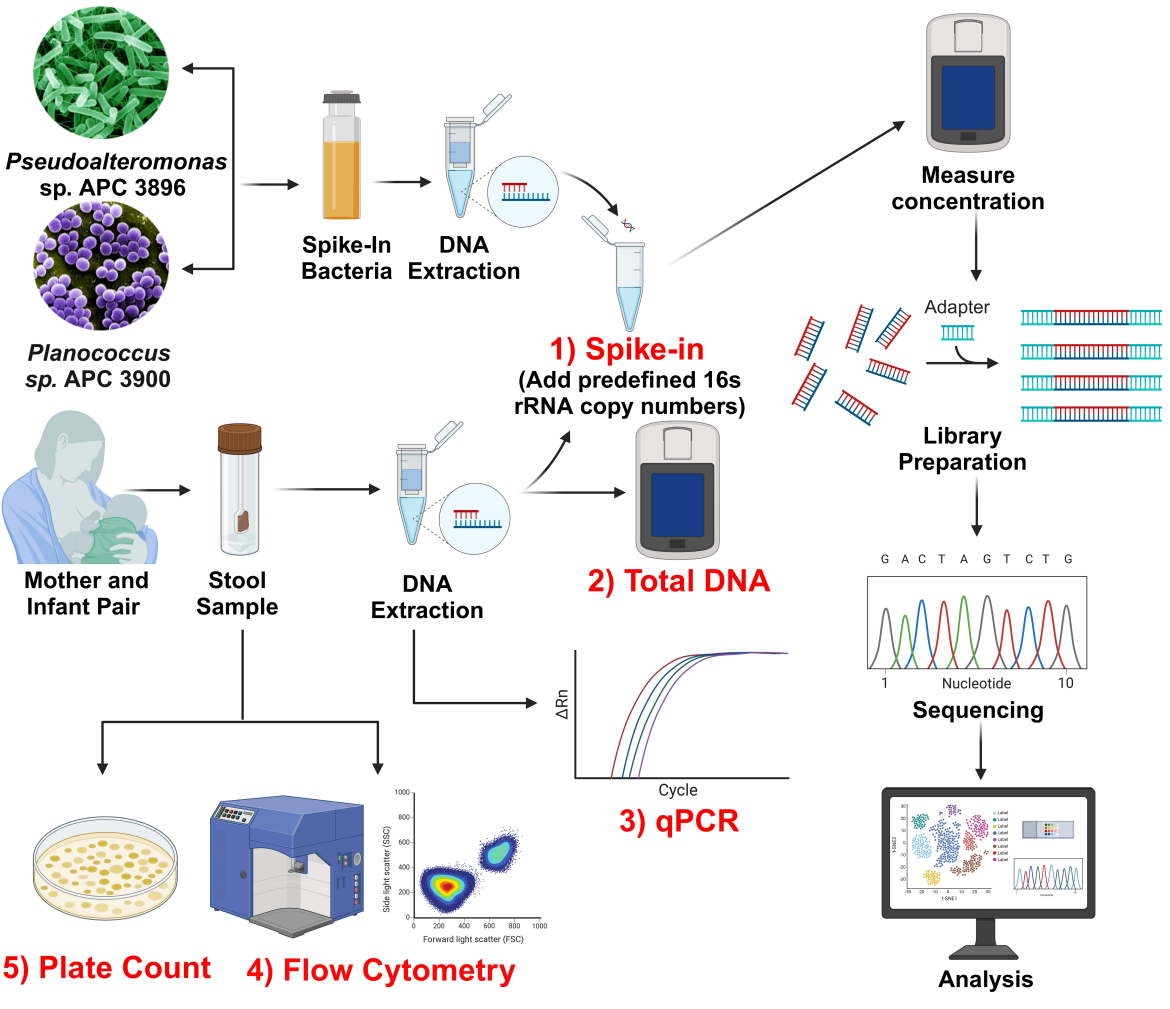
Overview of the proposed DNA spike-in procedure, compared with other quantification methods.

Figure 1 provides an overview of the DNA spike-in quantification method and its comparison with other alternative approaches. The initial step in the DNA spike-in procedure involved the extraction of DNA from the spike-in bacteria *Pseudoalteromonas* sp. APC 3896 and *Planococcus* sp. APC 3900, as well as from maternal and infant fecal samples. Accurate documentation of the mother and infant sample weights and elution buffer volumes during DNA extraction is crucial [Supplementary Table 1]. Following DNA extraction, a specified quantity of spike-in DNA was added to each sample's DNA, after which library preparation was undertaken. Given the predetermined number of rRNA copies added, this served as a back-normalization factor for accurately determining the absolute abundance of all other organisms present within the sample.

### The spike-in bacteria correlate well with microbial loads

**Figure 2:**
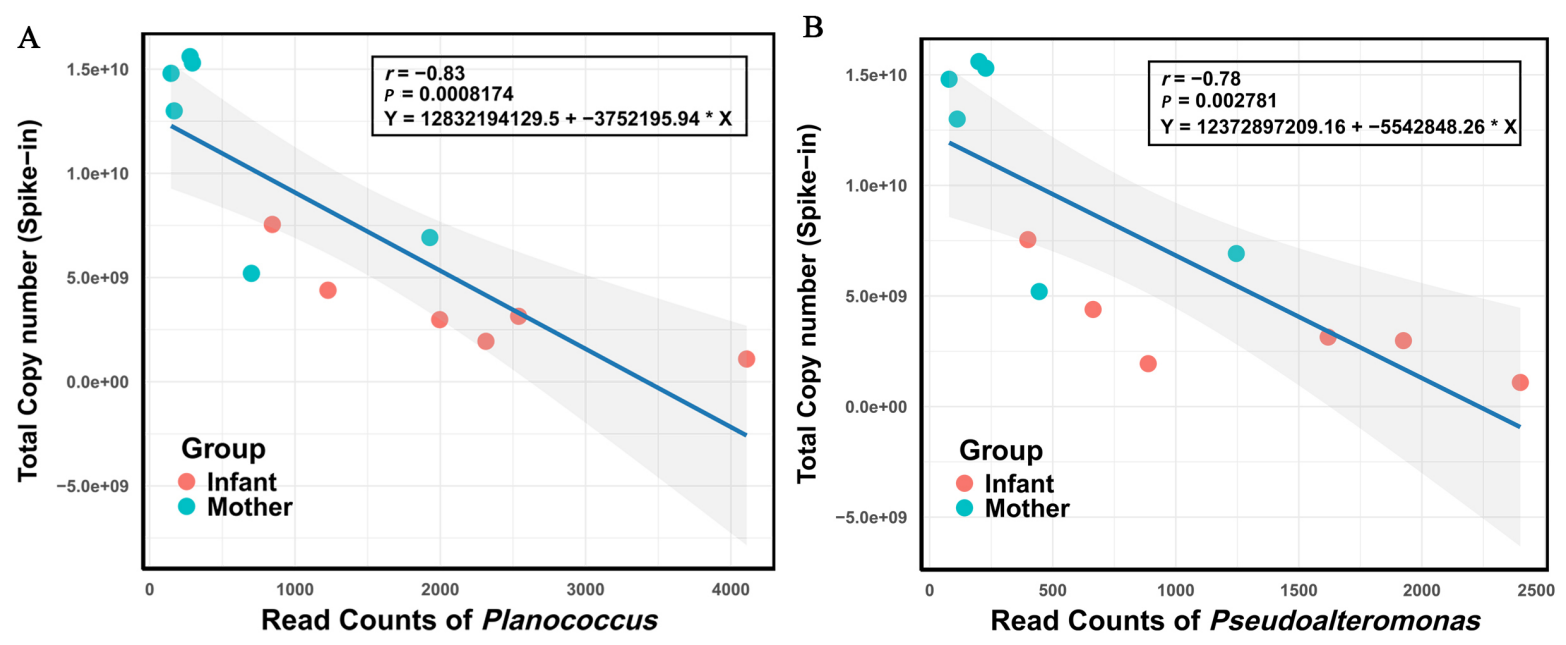
Correlation analysis of read counts of the two spike-in bacteria and the microbial loads from spike-in. The correlation analysis was performed using Pearson’s correlation coefficient. (A) The line chart illustrates the read counts of *Planococcus* as a function of microbial load from spike-ins across different samples; (B) The line chart illustrates the read counts of *Pseudoalteromonas* as a function of microbial load from spike-ins across different samples.

To validate the accuracy and reliability of the DNA spike-in method for estimating microbial loads, we examined the correlation between the spike-in bacterial read counts and the total microbial loads across different samples. Figure 2 illustrates the relationship between the read counts of the spike-in bacteria *Pseudoalteromonas* and *Planococcus* and the total copy number obtained from the spike-in as calculated from the weight of DNA added from the sample. The analysis shows an inverse correlation between the read count assigned to a spike-in operational taxonomic unit (OTU) and the total copy number from the spike-in method. For *Planococcus*, the correlation coefficient was *r* = -0.83, indicating a strong negative correlation (*P* = 0.0008174). Similarly, for *Pseudoalteromonas*, the correlation coefficient was found to be *r* = -0.78 (*P* = 0.002781). The close similarity between the two *r* values suggests that the spike-in method results in a consistent relationship with microbial load across different spike-in bacterial strains, although they are of different gram types. While the correlation for *Planococcus* was found to be slightly stronger than that for *Pseudoalteromonas*, both values confirm the effectiveness of the spike-in method for accurately quantifying microbial load.

### Spike-in comparison with other absolute quantification approaches (Flow Cytometry, Plate Count, qPCR, and Total DNA)

As shown in Figure 1A, flow cytometry produced significantly higher microbial cell counts compared to plate count (*P* = 0.01), spike-in (*P* = 0.002), and qPCR (*P* = 0.002). Plate count also yielded significantly higher counts than spike-in (*P* = 0.016) and qPCR (*P* = 0.036). No statistically significant difference was observed between spike-in and qPCR (*P* = 0.07), indicating general agreement between these two methods. Overall, the methods followed a descending trend in estimated bacterial load: Flow Cytometry > Plate Count > qPCR ≈ Spike-in.

When mother and infant groups were analyzed separately, significant differences in total microbial cell counts were detected using qPCR (*P* < 0.001) and spike-in quantification (*P* = 0.005), but not with flow cytometry or plate count. This highlights the higher sensitivity of DNA-based methods in detecting group-specific microbial load differences and further underscores the consistency between qPCR and spike-in results.

**Figure 3:**
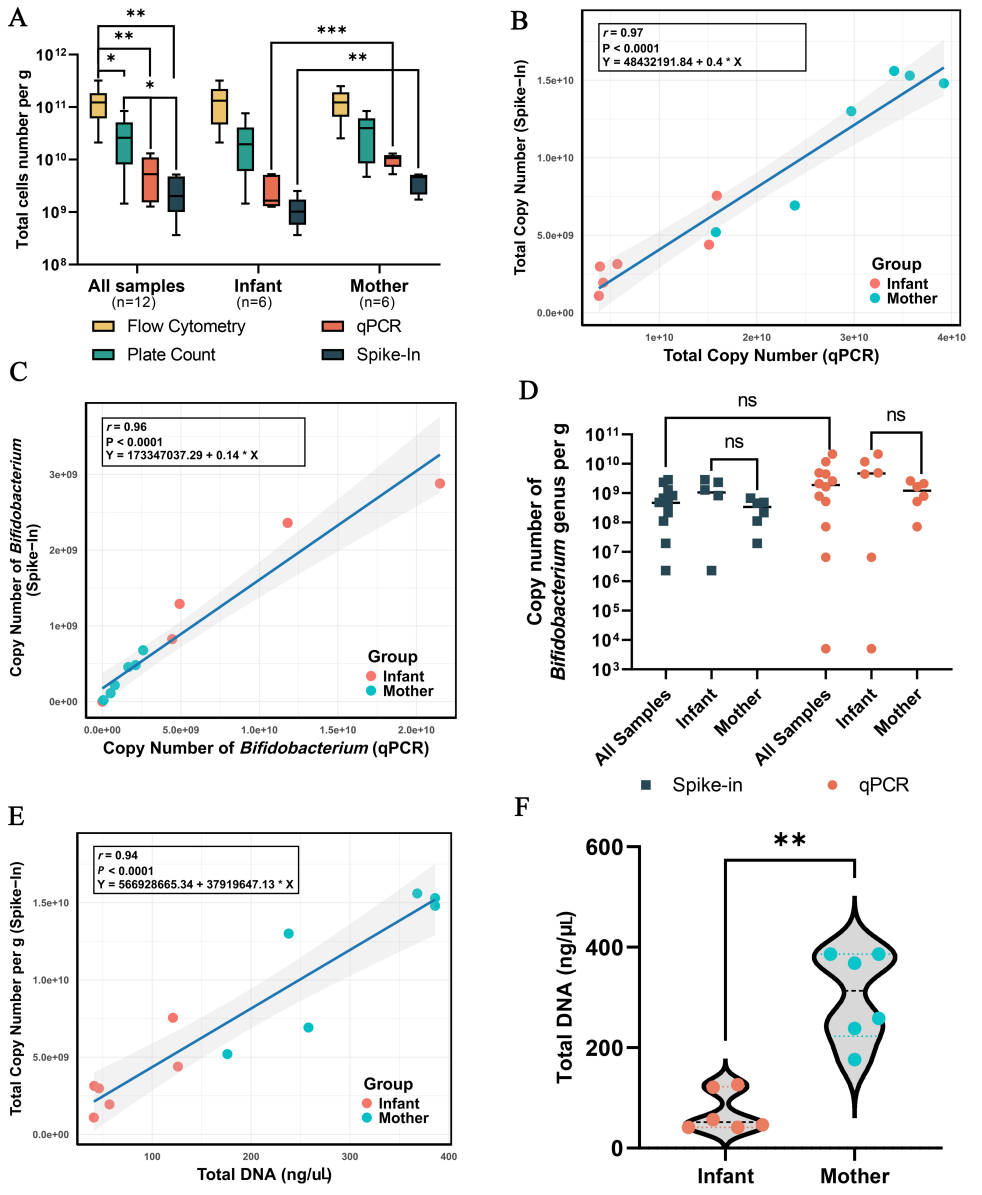
(A) Comparison of microbial cell counts (cells per gram feces) by using flow cytometry, plate count, qPCR, and Spike-in. Statistical significance between methods was analyzed using one-way ANOVA with Welch’s correction, followed by Games–Howell post hoc tests. Significance levels are indicated as *P < 0.05; **P < 0.01; ***P < 0.001; (B) Comparing spike-in quantification and qPCR from total 16s rRNA copy number and the copy number of *Bifidobacterium* genus; (C) Correlation analysis of spike-in and qPCR methods for measuring total copies and copy number of *Bifidobacterium* genera in mother and infant samples; (D) Significance analysis of copy number measurements of *Bifidobacterium* genera using both spike-in and qPCR methods in mother and infant samples; (E) Correlation Analysis between spike-in and total DNA. The correlation analysis of (B), (C), and (E) was performed using Pearson’s correlation coefficient; (F) Significance analysis between mother and infant groups by using total DNA measurements. qPCR: quantitative PCR.

A strong positive correlation was observed between qPCR and spike-in results across individual samples, with Pearson's correlation coefficient calculated as *r* = 0.97 (*P* < 0.0001) [Figure 3B]. This indicates a high degree of agreement between these two methods. Furthermore, total microbial load was significantly greater in the mother group compared to the infant group (*P* < 0.001), consistent across both qPCR and spike-in analyses.

To validate the reliability of the spike-in approach, genus-specific analysis using *Bifidobacterium*-specific primers in qPCR was performed. The results revealed a similarly strong correlation (*r* = 0.96, *P* < 0.0001) between *Bifidobacterium* copy numbers obtained via qPCR and spike-in quantification [Figure 3C]. Both methods showed that infants tended to have a higher abundance of *Bifidobacterium* than mothers, though this difference was not statistically significant [Figure 3C and D].

To compare bacterial loads in infant and mother fecal samples, we quantified the total DNA content. A strong positive correlation was observed between total DNA content and the spike-in method (*r* = 0.94, *P* < 0.001) [Figure 3E], demonstrating the reliability of the spike-in approach for estimating microbial loads. Consistent with findings from both the spike-in and qPCR methods, the total DNA content was significantly higher in samples from mother than infants (*P* = 0.002) [Figure 3F].

### Comparing relative and absolute abundance via spike-in

**Figure 4:**
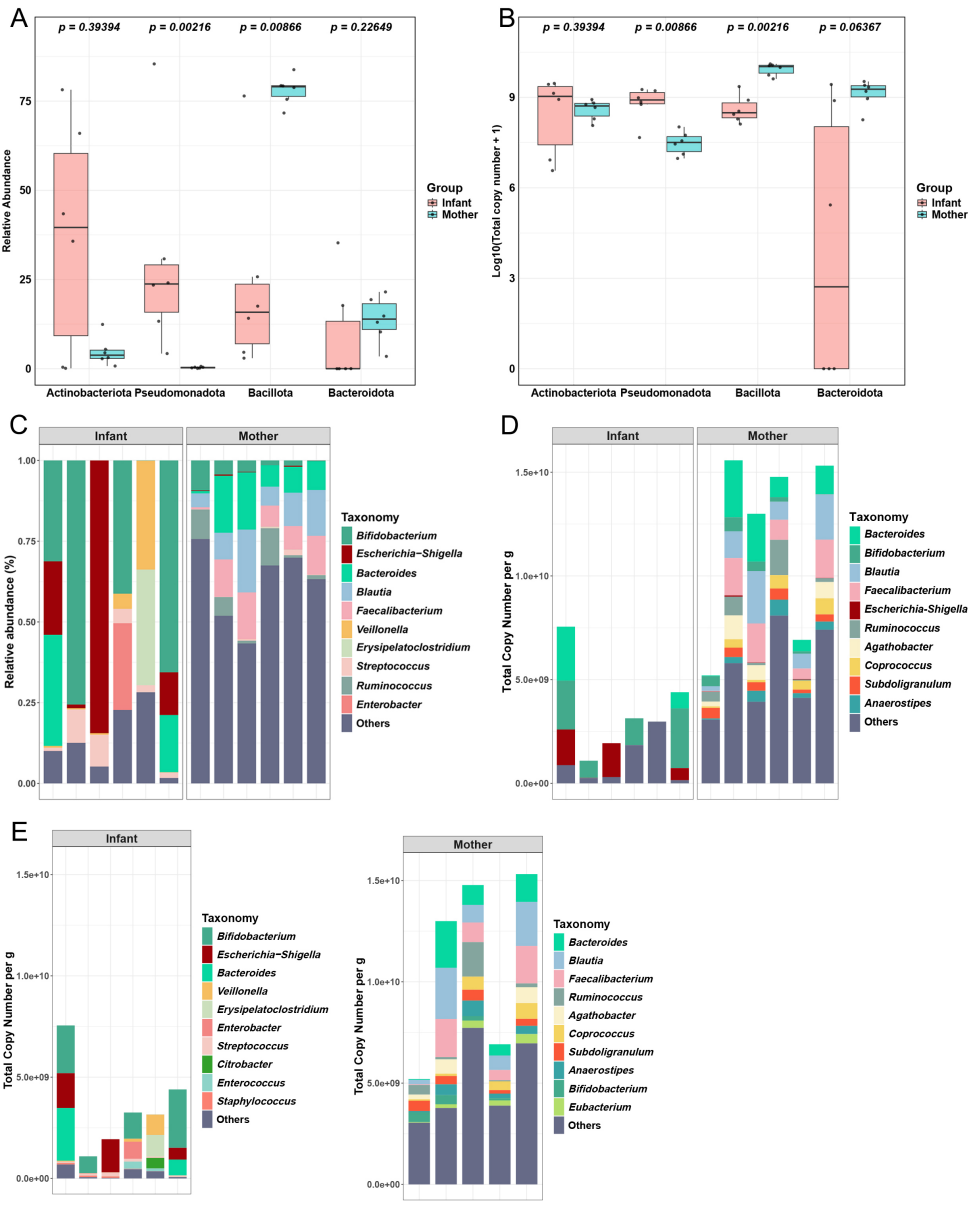
Genus-level composition of top 10 fecal microbiome members in mother and infant groups. (A) and (B) Taxonomic differential abundance analysis (Top four Phylum level). Statistical significance was analyzed using the Wilcoxon test. (A) Based on relative abundance; (B) Based on the total copy number of Spike-in; (C) Relative abundance based on the percentage of the top 10 genera; (D) Absolute abundance based on the copy number (per gram of feces) of the top 10 genera by using spike-in. The calculation of the top 10 microbial genera is based on the summation of genus copies from both mothers and infants, sorted accordingly; (E) The calculation of the top 10 microbial genera is based on the summation of genus copies separately from mothers and infants, and subsequently sorted.

To evaluate the impact of spike-in on microbiome analysis outcomes, we examined its effect on phylum and genus abundance profiles in the mother-infant pairs. In the top four phyla, significant differences were observed in the microbial profiles of infants and mothers [Figure 4A and B]. Pseudomonadota showed a significantly higher abundance in infants compared to mothers (relative: *P* = 0.0022; absolute: *P* = 0.0087), while Bacillota was more abundant in mothers in both analyses (relative: *P* = 0.0087; absolute: *P* = 0.0022). Actinomycetota and Bacteroidota did not display significant differences in either relative or absolute abundance, though Bacteroidota showed a trend toward higher abundance in infants in the absolute analysis (*P* = 0.0637).

To better compare the impact of the spike-in method on gut microbiota analysis, we selected the top 10 genera based on relative abundance and the top 10 genera obtained using the spike-in method. After using the absolute quantitative analysis with spike-in, the rankings of several genera within the top 10 in relative abundance changed significantly [Figure 4C and D]. For example, the rankings of the genera *Bifidobacterium*, *Escherichia-Shigella*, and *Bacteroides* shifted notably. Additionally, some genera, such as *Veillonella*, *Streptococcus*, and *Enterobacter*, that were within the top 10 in relative quantification are no longer in the top 10 in absolute quantification. Conversely, some genera, such as *Agathobacter*, *Coprococcus*, *Subdoligranulum*, and *Anaerostipes*, have entered the top 10 in absolute quantification.

Additionally, as observed in Figure 4D, the absolute quantification analysis revealed significant differences in microbial loads between mothers and infants. This disparity affected the top 10 genera in the infant group, which had lower microbial loads. To address this variation, we separately calculated the top 10 genera for the infant and mother groups. Upon independent calculation, we found that the top 10 rankings for the mother group remained largely unchanged, with a few exceptions [Figure 4E]. *Bifidobacterium* dropped from the second to the ninth position in abundance, and *Escherichia-Shigella* was no longer within the top 10. Notably, these two genera were the first and second most abundant genera in the infant group.

Interestingly, when performing absolute quantification to independently compute the top 10 genera within the infant group, the results show significant similarity to those obtained through relative quantification. *Veillonella*, *Erysipelatoclostridium*, *Streptococcus*, and *Enterobacter* have reappeared among the top 10 genera. Moreover, in absolute quantification analysis, the independent calculation of the top 10 genera within the mother group closely resembles the overall top 10 genera calculated across all samples. Key differences in genus rankings between relative and absolute quantification, including group-specific shifts in infants and mothers, are summarized in Supplementary Table 2, which facilitates clearer comparison across quantification strategies.

This highlights that in relative abundance analysis, samples with low microbial loads significantly influence the ranking of the top 10 genera. This effect arises from the assumption that all samples contribute equally to 100%. In contrast, in absolute quantification analysis, groups with high microbial loads exert considerable influence on the ranking of the top 10 genera across two or more groups, potentially obscuring true gut microbiota dynamics. Therefore, using absolute quantification methods to separately calculate the top 10 or even top 20 genera by group enables a more precise analysis of gut microbiota.

### Taxonomic differences of top 10 by relative abundance and absolute abundance

**Figure 5:**
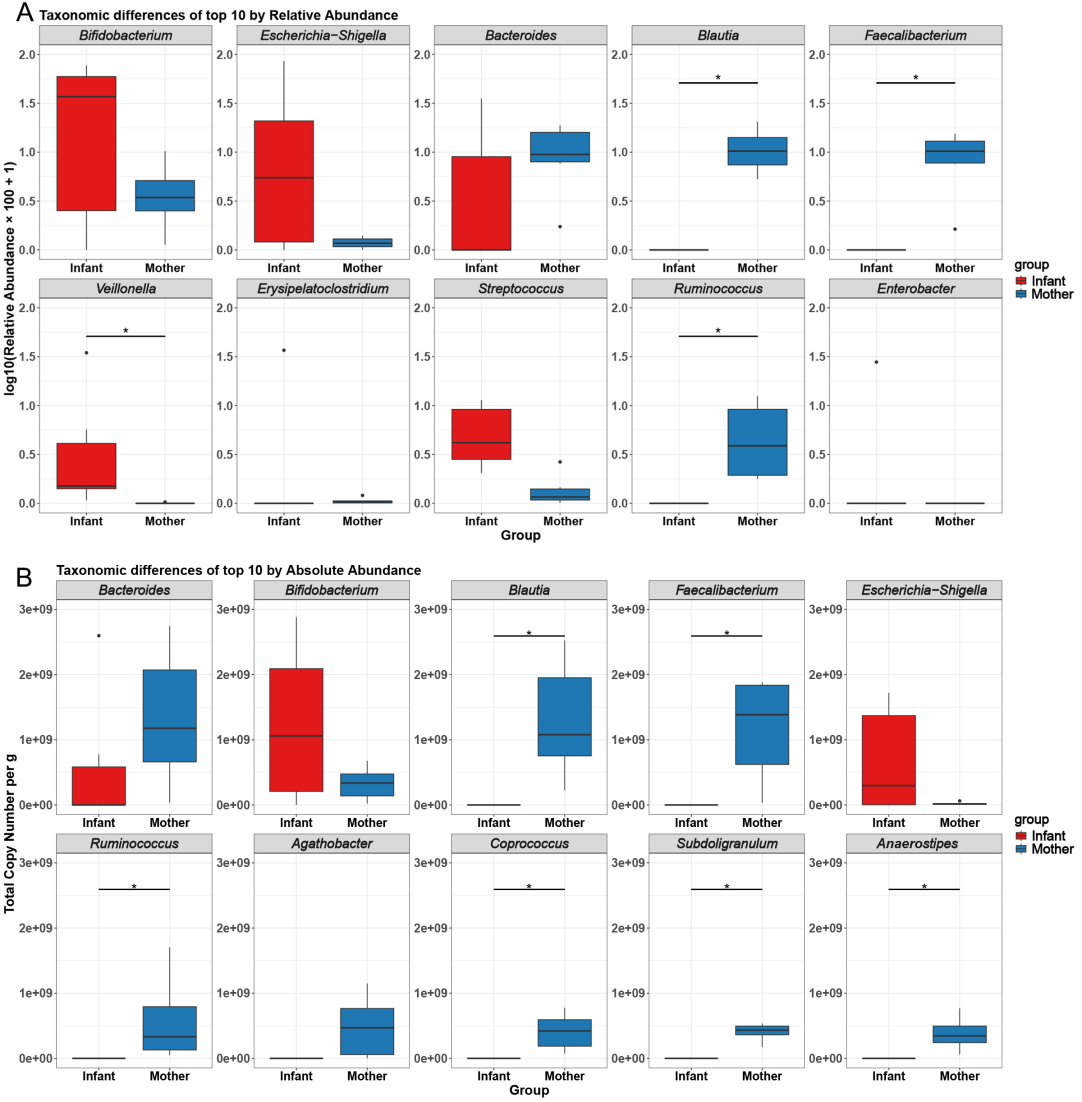
Differential abundance analysis of the top 10 genera in mother and infant groups. Statistical significance was analyzed using the Wilcoxon test. (A) Taxonomic differences of the top 10 genera by using relative abundance; (B) Taxonomic differences of top 10 genera by using absolute abundance. ^*^P < 0.05.

To assess the differential abundance of the top 10 genera based on relative and absolute quantification with spike-in, we conducted a differential abundance analysis using both methodologies [Figure 5]. In the relative abundance analysis, significant differences were observed between the mother and infant groups for the genera *Blautia*, *Faecalibacterium*, *Veillonella*, and *Ruminococcus*. However, after applying the spike-in absolute quantification method, significant differences were identified for the genera *Blautia*, *Faecalibacterium*, *Ruminococcus*, *Coprococcus*, *Subdoligranulum*, and *Anaerostipes*.

### α and β diversity

**Figure 6:**
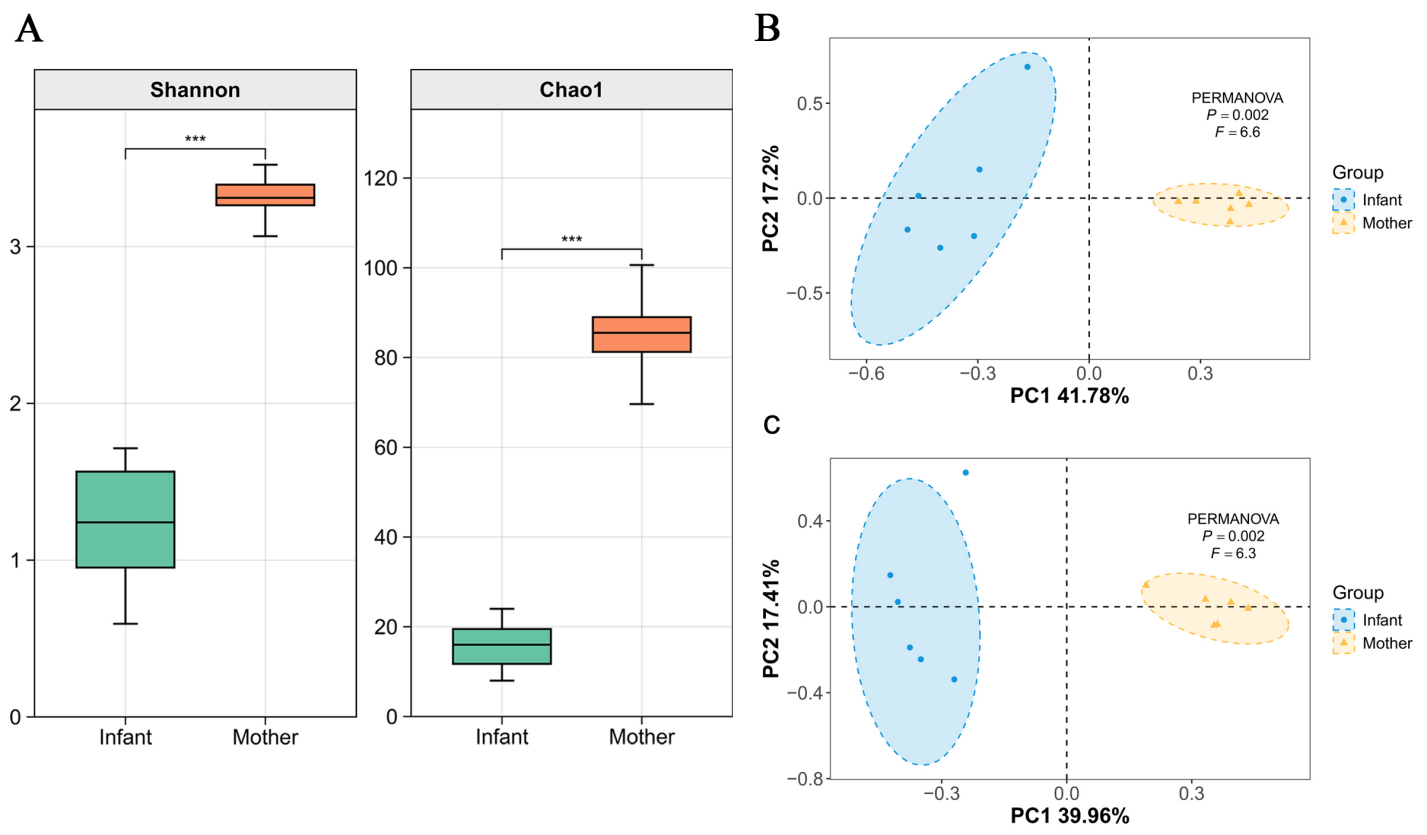
Effect of spike-in on the α diversity and *β*-diversity analysis. (A) α diversity indicated by the Shannon index and Chao1 index; (B) β-diversity Principal coordinates analyses (PCoA, Bray-Curtis’ dissimilarities) from relative abundance analysis; (C) *β*-diversity from spike-in analysis. ^***^P < 0.001.

Figure 6 presents the α- and β-diversity among the mother and infant pairs. The α-diversity, as measured by the Shannon index and Chao1 index, shows no difference between the relative analysis and the spike-in absolute quantification method [Figure 6A]. However, the indices indicate that the richness and evenness of the microbiome in the mother group are significantly higher than those in the infant group.

The Principal Coordinates Analysis (PCoA) plots illustrate the β-diversity found among the mother and infant pairs. In the PCoA plot derived from the relative abundance analysis [Figure 6B], the analysis shows a statistically significant separation between the mother and infant groups (*P* = 0.02, *F* = 6.6). Similarly, the PCoA plot from the absolute abundance analysis using spike-in [Figure 6C] shows a statistically significant difference between the groups (*P* = 0.02, *F* = 6.3). Despite the slight differences in the variance explained by PC1 and PC2, both PCoA plots consistently demonstrate significant separation between the mother and infant microbiomes.

## DISCUSSION

The purpose of this study was to evaluate different methods for absolute quantitation of bacterial loads in microbiomes with a view to proposing a method that would allow for analyzing large sample sets in our ongoing studies^[37]^. We propose using spike-in bacterial DNA to calibrate intestinal microbiome profiles to actual microbial loads. *Pseudoalteromonas* sp. APC 3896 and *Planococcus* sp. APC 3900 were employed as spike-in bacteria, demonstrating their suitability for comprehensive gut microbiome profiling. These two bacteria are typically absent in mammalian intestinal microbiomes, making them effective reporters of true microbial load. Incorporating spike-in bacteria offers a novel perspective to gut microbiome profiling, enhancing the standard relative composition analysis.

Although this study utilized only two spike-in strains and relied on 16S rRNA gene sequencing, the complete absence of *Pseudoalteromonas* and *Planococcus* genera in human gut microbiomes justifies the use of genus-level abundance as a reliable proxy for species-level quantification. This rationale is supported by both our current cohort data and ongoing analyses of large-scale metagenomic datasets. Moreover, when applying this method in metagenomic sequencing, it becomes possible to perform a dual-level validation: the abundance of *Pseudoalteromonas* and *Planococcus* can be cross-verified at both the genus and species levels, further confirming the accuracy and consistency of the spike-in approach.

While our marine-derived spike-in sequences minimize overlap with gut microbes, this does not eliminate the risk of differential amplification due to GC content, secondary structure, or mismatches at primer binding sites. These are intrinsic to 16S amplification, not the spike-in itself. However, because all samples receive identical spike-in DNA and undergo the same library preparation, the bias - though present - is consistent across samples, allowing meaningful cross-group comparisons.

The natural variation in intestinal microbial loads is a significant and potentially clinically important feature that is often overlooked in standard protocols. Technically, adding spike-in DNA directly to the sample DNA can remove any potential errors introduced due to lysis of the spike-in bacteria, as might happen when using whole cells. For samples that have already been extracted and sequenced, the spike-in method can be applied starting from library preparation instead of extraction from the beginning to investigate the influence of microbial loads, especially when sample quantities are very low.

In this study, we observed significant differences in the estimation of microbial loads between flow cytometry and the spike-in method, which may be attributed to the overestimation of cells in flow cytometry and variations in DNA extraction efficiency.

When using the Bacterial Viability and Counting Kit for flow cytometry, sample preparation steps like sonication and vortexing may cause foam formation and microbubbles, potentially leading to an overestimation of cell counts^[38-40]^. Different levels of overestimation have been observed when measuring different types of bacteria using flow cytometry, which was consistent with our current results^[14]^.

Overall, plate count resulted in bacterial counts approximately 7-8 times less than those obtained through flow cytometry, suggesting that the actual bacterial load likely falls within this range. While it is acknowledged that not all bacteria grow on the YCFA medium, our prior experience indicates that the majority of bacterial taxa can be successfully cultivated using this method^[22]^.

Notably, DNA extraction variability is a major factor contributing to experimental variation, leading to a reduced relative abundance of gram-positive bacteria in the samples. This issue has also been observed in numerous previous studies^[41-43]^. We used bead-beating for DNA extraction to ensure effective cell disruption, especially for hard-to-lyse microbes like gram-positive bacteria^[43-45]^.

The proposed DNA spike-in quantification method offers several advantages. Compared to flow cytometry, the spike-in method is faster, easier to perform, and provides better sample reproducibility. Flow cytometry, on the other hand, is labor-intensive and time-consuming, with optimal counting accuracy only within the range of 10^5^ to 10^7^ bacteria per mL^[14]^, which may limit its reliability in low-biomass or antibiotic-affected samples such as infant feces. Commercial products such as the ZymoBIOMICS**^TM^** Spike-in Control (Zymo Research) have adopted a related strategy for absolute quantification, using non-native bacterial cells added directly to fecal samples prior to DNA extraction. In contrast, our approach adds purified spike-in DNA after extraction, which avoids biases introduced by variable cell lysis efficiency. This also enables absolute quantification for previously extracted or archived samples, which would be incompatible with cell-based spike-in strategies. Together, these features highlight the methodological flexibility of spike-in–based quantification, as reflected in both commercial and research settings.

A limitation of the spike-in method is its reliance on copy number as the measurement standard, which can lead to the propagation of PCR amplification errors from the spike-in bacterium to other taxonomic units^[46]^. This occurs because different organisms have varying 16S rRNA gene copy numbers (GCNs), causing sequence variant counts to be biased toward clades with higher GCNs^[47]^. While this primarily affects comparisons between different genera, it has less impact on comparisons within the same genus, as genera-specific amplification errors tend to cancel out^[15]^.

To mitigate this effect, using multiple spike-in bacteria with fixed copy numbers across samples and averaging or summing their counts can be more effective. In this study, we selected two phylogenetically distinct spike-in strains (Gram-positive and Gram-negative) to average out potential taxon-specific amplification effects, thereby enhancing the robustness of absolute quantification. Furthermore, based on both previous studies and manufacturer guidelines (ZymoBIOMICS Spike-in Control), it is recommended that spike-in DNA should constitute between 0.1% and 10% of the total microbial DNA. Maintaining the spike-in proportion within this range helps ensure sufficient detection sensitivity while minimizing potential perturbations to the native microbial community structure. In our study, spike-in concentrations were adjusted based on preliminary testing to consistently fall within this optimal range across all samples. These tests showed that adding spike-in DNA corresponding to approximately 10^6^-10^7^ copies per sample typically yielded a final proportion within the 0.1%-10% range across a variety of microbial loads. This strategy further reduces the likelihood that amplification biases from spike-in bacteria significantly affect the accuracy of absolute quantification.

Additionally, single-copy housekeeping genes (e.g., *rpoB*, *recA*) offer a theoretically more stable alternative for cell-based quantification, but their limited phylogenetic coverage and the need for taxon-specific primer design make them less suitable for high-throughput, whole-community analyses. Future strategies may combine spike-in normalization for total load calibration, targeted detection of single-copy genes for key taxa, and metagenomic approaches to avoid PCR amplification bias altogether.

Another limitation arises from the detection threshold of 16S rRNA sequencing, where low-abundance taxa (approximately fewer than 10^5^ cells) may be reported as having zero abundance due to insufficient sequencing depth^[48]^. In this study, one infant sample exhibited a zero abundance of *Bifidobacterium* in the 16S rRNA sequencing results, while qPCR analysis detected approximately 10^3^ copies, reflecting the higher sensitivity of qPCR. Consequently, using DNA spike-in with 16S rRNA sequencing may lead to an underestimation of the total microbial load. However, employing DNA spike-in with shotgun metagenomic sequencing can provide a more accurate and comprehensive quantification of microbial abundance.

Among the various spike-in absolute quantification methods, using extracted DNA as a spike-in instead of directly adding cells to fecal samples offers significant advantages. This approach eliminates biases associated with cell lysis efficiency and DNA extraction recovery^[49,50]^, ensuring more reliable quantification. Additionally, for samples with pre-extracted DNA, quantification can begin directly from the DNA, bypassing the need for additional sample preparation steps. Compared to spike-in synthetic DNA sequences, which may introduce artificial background noise or biases in bioinformatic analyses during sequencing^[18]^, using authentic biological DNA, such as marine bacterial DNA, better mimics human gut microbiome DNA. This reduces potential interference caused by sequence discrepancies and enhances the accuracy of microbial community profiling.

The use of copy number as a standard can result in amplification efficiency errors during 16S rRNA sequencing, affecting total copy number estimation^[51]^. This issue is particularly pronounced in samples with a high abundance of high-copy-number bacteria. However, this impact should be minimized in shotgun sequencing, as it involves shearing DNA rather than amplification^[51,52]^.

The observed shifts in genus rankings between relative and absolute quantification [Figure 4C-E] are driven by both total microbial load differences and individual taxon variation, with total load playing the primary role. This effect is particularly evident between infant and mother samples, where mothers had significantly higher total bacterial counts, amplifying the contribution of dominant maternal genera in absolute terms. For instance, while *Bifidobacterium* appeared dominant in infants based on relative abundance, its absolute copy number was comparable between infants and mothers, indicating a load-driven distortion. In contrast, *Escherichia-Shigella* remained significantly more abundant in infants even after normalization, reflecting true taxon-specific enrichment. These results underscore the importance of absolute quantification in disentangling microbial abundance from compositional bias. Without adjusting for total load, key ecological signals may be obscured or misrepresented in relative abundance–based analysis.

When comparing relative abundance with absolute abundance after spike-in processing, it was found that *Veillonella*, *Streptococcus*, and *Enterobacter* are no longer among the top 10 in the absolute quantification. *Veillonella*'s biofilm-forming ability^[53]^, *Streptococcus*’s transformation capability^[54]^, and *Enterobacter*'s drug efflux mechanisms^[55]^ confer antibiotic resistance to these genera. Consequently, when participants take antibiotics, the total number of bacterial cells decreases^[56]^, and antibiotic-resistant genera become more prominent in relative quantification analyses. This finding is consistent with previous studies^[16]^.

The F value in both analyses indicates the ratio of the variance between the groups to the variance within the groups^[57]^. The slightly higher *F* value in the relative abundance analysis (*F* = 6.6) compared to the spike-in absolute abundance analysis (*F* = 6.3) suggests that the relative abundance analysis exhibits a slightly greater distinction between the mother and infant microbiomes. This difference in *F* values may be attributed to the inherent variability and potential biases in relative abundance measures, which can sometimes amplify differences between groups. In contrast, the spike-in method aims to reduce such biases by providing an absolute quantification, potentially leading to a more accurate but slightly lower *F* value.

This interpretation is further supported by taxonomic patterns observed in Figure 4, where high-biomass maternal taxa such as *Blautia* and *Coprococcus* and infant-associated taxa like *Escherichia-Shigella* and *Bifidobacterium* largely drove the separation in both relative and absolute β-diversity plots. Genera like *Veillonella*, although prominent in relative terms, were low in absolute abundance and contributed less to the spike-in–based analysis. The similar PCoA clustering patterns and consistent variance explained across PC1 and PC2 further support that absolute *β*-diversity reflects the same underlying biological differences - while reducing distortion from low-abundance taxa.

However, the use of marine-derived spike-in strains presents a limitation in broader microbiome research. While these strains are ideal for human gut studies due to their absence in mammalian microbiomes, they may be less suitable for environmental or indoor microbiomes, where trace marine DNA is more likely to be present. In such contexts, the spike-in signal may be confounded by endogenous microbial DNA, reducing quantification accuracy. Alternative strategies, such as qPCR, have been more commonly adopted for absolute quantification in these settings^[58,59]^. Future adaptations of this method may include selecting habitat-specific spike-ins to improve applicability across different ecosystems.

Our data suggest that the spike-in method offers a strong balance between practicality and accuracy, particularly in 16S rRNA-based studies involving low-biomass or compositionally variable samples. While not a universal standard, it is especially well suited for microbiome comparisons in mother-infant cohorts, intervention studies, and archived samples with limited input. In contrast, for environmental microbiomes, adapting spike-in strains may be necessary. Overall, the method provides a scalable and accessible approach when total microbial load is a key variable of interest.

In conclusion, by spiking exogenous bacterial DNA from *Pseudoalteromonas* sp. APC 3896 and *Planococcus* sp. APC 3900 into extracted fecal DNA, we demonstrated that DNA spike-in can reliably normalize microbial load differences and enable accurate comparisons of community profiles. Even under fluctuating microbial biomass, this approach effectively stabilized quantification across samples. The application of spike-in-based absolute quantification revealed notable shifts in the rankings of predominant genera, underscoring its importance in uncovering biologically meaningful variation otherwise masked in relative abundance analyses.

This method also captured clearer distinctions in β-diversity between mother and infant microbiomes, supporting its utility for detecting biologically relevant community differences. Overall, the spike-in strategy offers a practical, scalable, and reproducible alternative to traditional relative abundance measures - improving accuracy in quantifying microbial dynamics and advancing our understanding of microbiome ecology in health and disease.

## DECLARATIONS

### Availability of data and materials

The data that support the findings of this study are available from the corresponding author upon reasonable request.

### Copyright

© The Author(s) 2025.
